# Identification of the Plant Compound Geraniin as a Novel Hsp90 Inhibitor

**DOI:** 10.1371/journal.pone.0074266

**Published:** 2013-09-16

**Authors:** Antonio Vassallo, Maria Carmela Vaccaro, Nunziatina De Tommasi, Fabrizio Dal Piaz, Antonella Leone

**Affiliations:** 1 Dipartimento di Scienze, Università degli Studi della Basilicata, Potenza, Italy; 2 Dipartimento di Farmacia, Università degli Studi di Salerno, Fisciano, Italy; Technische Universitaet Muenchen, Germany

## Abstract

Besides its function in normal cellular growth, the molecular chaperone heat shock protein 90 (Hsp90) binds to a large number of client proteins required for promoting cancer cell growth and/or survival. In an effort to discover new small molecules able to inhibit the Hsp90 ATPase and chaperoning activities, we screened, by a surface plasmon resonance assay, a small library including different plant polyphenols. The ellagitannin geraniin, was identified as the most promising molecule, showing a binding affinity to Hsp90α similar to that of 17-(allylamino)-17-demethoxygeldanamycin (17AGG). Geraniin was able to inhibit in vitro the Hsp90α ATPase activity in a dose−dependent manner, with an inhibitory efficiency comparable to that measured for 17-AAG. In addition, this compound compromised the chaperone activity of Hsp90α, monitored by the citrate synthase thermal induced aggregation assay. Geraniin decreased the viability of HeLa and Jurkat cell lines and caused an arrest in G_2_/M phase. We also proved that following exposure to different concentrations of geraniin, the level of expression of the client proteins c-Raf, pAkt, and EGFR was strongly down−regulated in both the cell lines. These results, along with the finding that geraniin did not exert any appreciable cytotoxicity on normal cells, encourage further studies on this compound as a promising chemical scaffold for the design of new Hsp90 inhibitors.

## Introduction

Heat shock protein 90 (Hsp90) is a highly conserved molecular chaperone that modulates cellular homeostasis and environmental stress responses by interacting with more than 200 different proteins, known as Hsp90 client proteins, to facilitate their correct folding and biological activity. Besides assisting proper protein folding and assembly, Hsp90 is also critical to target misfolded proteins for proteolytic degradation [1]–[3]. Most of the Hsp90 client proteins are involved in cell growth, differentiation and survival, and include kinases, nuclear hormone receptors, transcription factors and other proteins associated with almost all the hallmarks of cancer [4], [5]. Consistent with these diverse activities, genetic and biochemical studies have demonstrated the implication of Hsp90 in a range of diseases, also including cancer and allograft rejection [6]. Although Hsp90 is required in all cells, tumor cells are especially sensitive to Hsp90 inhibitors due to the critical role played by this chaperone in stabilizing several oncoproteins [7]. Inhibition of Hsp90 activity incapacitates simultaneously multiple client proteins, resulting in a blockade of multiple signaling pathways and, ultimately, providing a combinatorial attack to cellular oncogenic processes [8]. Because of the potential therapeutic use in multiple cancer indications, Hsp90 has emerged as an interesting target for the development of antitumor agents: thirteen new Hsp90 inhibitors are currently under evaluation at various stages of clinical trials [9].

Several natural product inhibitors of Hsp90 have been discovered targeting the ATPase binding site of the chaperone, such as geldanamycin and its semi−synthetic derivatives 17-(allylamino)-17-demethoxygeldanamycin (17-AAG) and 17-dimethylaminoethylamino-17-demethoxygeldanamycin (17-DMAG), radicicol and novobiocin [10]. However, despite the anti−tumorigenic and anti−angiogenic properties proved for the 17-AAG and 17-DMAG in *in vitro* and *in vivo* animal models, clinical trials have been only relatively successful [11], [12]. This failure uncovers the need to discover novel Hsp90 inhibitors based on diverse chemical skeletons and with superior chemotherapeutic properties for cancer treatment. Recently, several plant−derived small molecules have been discovered exhibiting inhibitory activity towards Hsp90, such as epigallocatechin gallate [13], gedunin [14], lentiginosine [15], celastrol [3] and deguelin [16].

**Figure 1:**
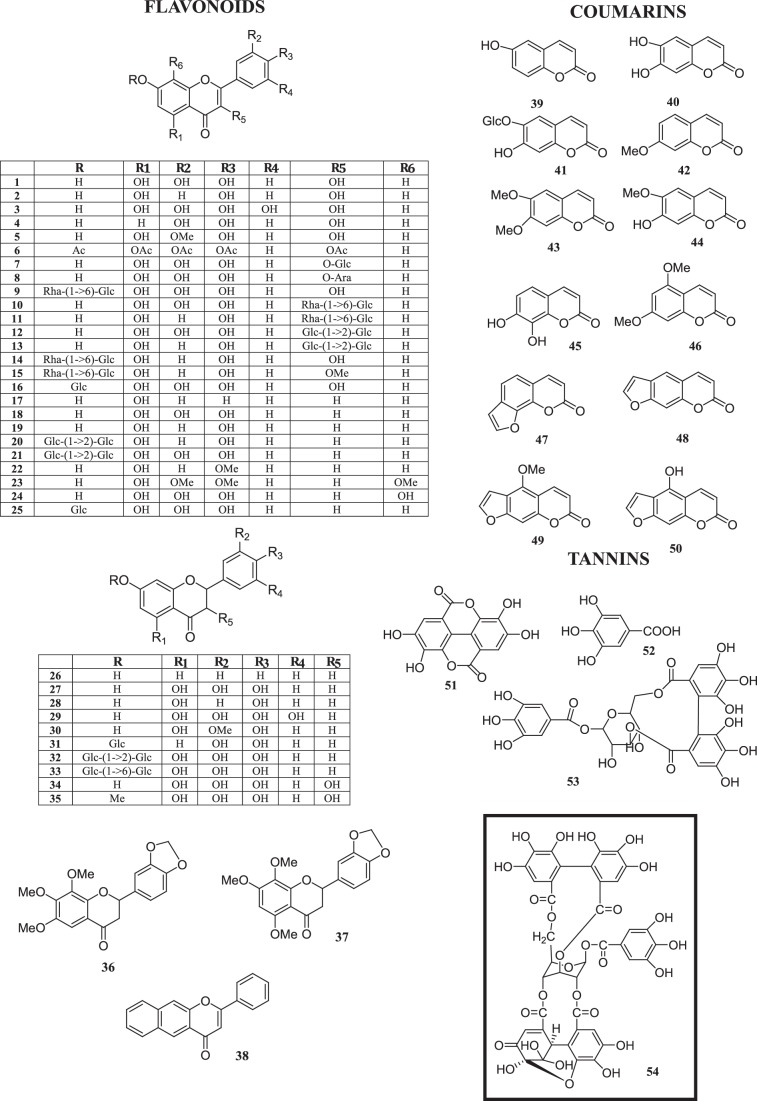
Structure of the 54 tested compounds. Geraniin structure (54) is evidenced.

With the view to identifying new potential Hsp90 inhibitors, we have used a surface plasmon resonance (SPR) assay to screen a small library including different phenolic compounds, such as flavonoids, tannins and coumarins (Figure 1). Among the different flavonoids and tannins that were able to bind Hsp90, we focused on the ellagitannin geraniin (compound **54** in Figure 1), the main polyphenolic compound in *Geranium thunbergii*, a medicinal plant used to treat diarrhea in Japan.

Ellagitannins belong to the hydrolyzable tannin group and occur in several food plants, such as raspberries, strawberries, blackberries, pomegranate, almonds, and walnuts [17]. *In vitro* and *in vivo* studies have demonstrated various biological activities of this class of compounds, including antioxidant [18], antiviral, antimutagenic, antimicrobial, and antitumor effects [19], suggesting that the consumption of ellagitannins might be beneficial to human health. Geraniin is a typical ellagitannin because it is composed entirely of common acyl units, such as galloyl, hexahydroxydiphenoyl (HHDP), and dehydrohexahydroxydiphenoyl (DHHDP) groups. Although variousstudies of geraniin have proved its antioxidant, antitumor, and antivirus properties [20], [21], its mechanism of action is still poorly characterized. Herein, we report the results of a panel of chemical and biological approaches that demonstrate that geraniin binds to Hsp90α and inhibits its ATPase activity, thus compromising the stability of some oncogenic client proteins.

Our results indicated that geraniin could represent an innovative scaffold for the design of new Hsp90 inhibitors interacting with its ATPase domain.

## Materials and Methods

### Materials

All the tested compounds belong to the plant-derived chemical library of the Department of Pharmacy, University of Salerno. Solvents (HPLC grade) were purchased from Romil (ROMIL Ltd, Cambridge, UK). All buffers were prepared with a Milli-Q water apparatus (Millipore, Bedford, MA, USA). Recombinant human Hsp90α was purchased at Assay Designs (Ann Arbor, MI, USA). Proteomic grade trypsin was purchased from Sigma-Aldrich (Sigma-Aldrich Co, St Louis, MO, USA). Anti-Hsp 70 (Abcam, Cambrige,UK), anti-Hsp 90α/ß (H-114) sc-7947, anti-Raf1 (C-12): sc-133, anti-Akt (G-5):sc-55523, anti-pAkt sc, anti-EGFR (1005): sc-03 antibodies were purchased from Santa Cruz Biotechnology (Santa Cruz Biotechnology, Inc., Delaware, CA, USA). Anti-actin antibody and the Hsp90 inhibitor 17-(Allylamino)-17-demethoxygeldanamycin (17-AAG) were purchased from Sigma-Aldrich.

### Surface Plasmon Resonance Analyses

SPR analyses were carried out according to our previously published procedures [22], [23]. Briefly, SPR analyses were performed using a Biacore 3000 optical biosensor equipped with research−grade CM5 sensor chips (GE Healthcare). Using this platform, two separate recombinant Hsp90α surfaces, a BSA surface and one unmodified reference surface were prepared for simultaneous analyses. Proteins (100 µg mL^−1^ in 10 mM CH_3_COONa, pH 5.0) were immobilized on individual sensor chip surfaces at a flow rate of 5 µL min^−1^ using standard amine−coupling protocols to obtain densities of 8–12 kRU. Polyphenols, as well as 17-AAG and hardwickiic acid (HA), were dissolved in 100% DMSO to obtain 4 mM solutions, and diluted 1∶200 (v/v) in PBS (10 mM NaH_2_PO_4_, 150 mM NaCl, pH 7.4) to a final DMSO concentration of 0.5%. Compounds concentration series were prepared as twofold dilutions into running buffer: for each sample, the complete binding study was performed using a six−point concentration series, typically spanning 0.025–1 µM, and triplicate aliquots of each compound concentration were dispensed into disposable vials. Binding experiments were performed at 25°C, using a flow rate of 50 µL min^−1^, with 60 s monitoring of association and 300 s monitoring of dissociation. Simple interactions were suitably fitted to a single−site bimolecular interaction model (A+B = AB), yielding a single *K*_D_. Sensorgram elaborations were performed using the BIAevaluation software provided by GE Healthcare.

### ATP Hydrolysis Inhibition

The assay was performed using the Discover RX ADP HunterTM Plus Assay kit, following the manufacturer’s instructions. ATPase reactions were carried out for 60 min at 40°C temperature in 100 mM Tris pH 7.4, 100 µM ATP and 40 nM Hsp90α in the presence of different concentrations of geraniin or 17-AAG. ADP generation was measured using a Perkin Elmer LS 55 fluorimeter (540 nm excitation and 620 nm emission). Fluorescence intensity values measured for Hsp90α without any compound were set at 100% of enzyme activity. The background reaction rate was measured in a reaction lacking enzyme or substrate and subtracted from the experimental rates.

### Citrate Synthase Aggregation Assay

Chaperone activity was evaluated by monitoring the thermal−induced aggregation of citrate synthase (CS) (0.075 µM), as reported elsewhere [24], in the absence or presence of a stoichiometric amount of Hsp90α and 0.3 µM ATP, and with or without a four−fold molar excess of each testing compound. Aggregation was initiated by unfolding CS incubating the protein in 40 mM HEPES–KOH, pH 7.5 at 43°C. Aggregation was monitored by measuring light scattering at right angles with the Perkin Elmer LS 55 fluorimeter in stirred and thermostatted quartz cells. Both the emission and excitation wavelengths were set at 500 nm, while the band–pass was 2 nm. Kinetics traces reported are the averages of two separate measurements.

### Limited Proteolysis

Limited proteolysis experiments were performed on recombinant Hsp90α at 37°C, PBS 0.1% DMSO, using trypsin or chymotrypsin as proteolytic agents; 30 µL of a 3 µM Hsp90α solution were used for each experiment. Binary complex Hsp90α/geraniin was formed by incubating the protein with a 5∶1 molar excess of geraniin at 37°C for 15 min prior to proteolytic enzyme addition. Both Hsp90 and Hsp90/geraniin complex were digested using a 1∶100 (w/w) enzyme to substrate ratio. The extent of the reactions was monitored on a time−course basis by sampling the incubation mixture after 5, 15, and 30 min of digestion. Samples were analyzed by MALDITOF/MS using a MALDI micro MX (Waters). Mass data were elaborated using the Masslynx software (Waters). Preferential hydrolysis sites on Hsp90α under different conditions were identified on the basis of the fragments released during enzymatic digestion.

### Cell Culture and Treatment

Jurkat and HeLa (epithelial carcinoma) cells, obtained from Cell Bank in GMP-IST (Genova, Italy) were maintained in RPMI 1640 medium or Dulbecco’s modified Eagle medium (DMEM), respectively, supplemented with 10% (v/v) FBS, 2 mM l−glutamine and antibiotics (100 U mL^−1^ penicillin, 100 µg mL^−1^ streptomycin) purchased from Invitrogen (Carslbad, CA, USA), at 37°C in humidified atmosphere with 5% CO_2_. To ensure logarithmic growth, cells were subcultured every two days. As control cells, human peripheral blood mononuclear cells (PBMC) were isolated from buffy coats of healthy donors (kindly provided by the Blood Center of the Hospital of Battipaglia, Salerno, Italy) by using standard Ficoll−Hypaque gradients. Freshly isolated PBMC contained 92.8±3.1% live cells. Proliferation of PBMC was induced by phytohemagglutinin (PHA) (10 µg mL^−1^). Tumor and PBMC cells were treated with different concentrations of geraniin or 17-AAG to evaluate their cytotoxic effects. Stock solutions of geraniin (50 mM in DMSO) and 17-AAG (10 mM in DMSO) were stored a −20°C in the dark and diluted just before addition to the sterile culture medium. In all the experiments, the final concentration of DMSO was 0.10% (v/v).

### Cell Proliferation and Viability

Jurkat (2×10^4/^well) and HeLa (5000/well) cells were seeded in triplicate in 96 well−plates and incubated for 24 h in the absence or presence of different concentrations of geraniin (between 0,5 µM to 50 µM) or 17-AAG (between 0,5 nM to 10 µM).

The number of viable cells was determined by using a [3–4,5-dimethyldiazol-2-yl]-2,5-diphenyl tetrazolium bromide (MTT, Sigma-Aldrich) conversion assay, according to the method described by Mosmann [25]. Briefly, following the treatment, 25 µL of MTT (5 mg/mL in PBS) was added and the cells were incubated for additional 3 h at 37°C. Thereafter, cells were lysed and suspended with 100 µL of buffer containing 50% (v/v) N,N-dimethylformamide, 20% SDS (pH 4.5). The absorbance was measured with a microplate reader (Titertek multiskan MCC7340, LabSystems, Vienna, VA, USA) equipped with a 620 nm filter. Cell population growth inhibition was tested by cytometric counting (trypan blu exclusion). IC_50_ values were calculated from cell viability dose−response curves and defined as the concentration resulting in 50% inhibition of cell survival, compared to control cells treated with 0.10% DMSO.

### Analysis of Cell Cycle and Hypodiploidy by Flow Cytometry

Cell DNA content was measured by propidium iodide (PI) incorporation into permeabilized cells, as described by Nicoletti et al. [26]. Briefly, the cells were harvested after geraniin or 17-AAG treatments were washed with cold PBS and incubated with a PI solution (0.1% sodium citrate, 0.1% Triton X-100 and 50 µg mL^−1^ of prodium iodide, Sigma-Aldrich, 10 µg/ml Rnase A) for 30 min at room temperature. Data from 10.000 events per sample were collected by a FACScalibur flow cytometer (Becton Dickinson, San José, CA) and cellular debris was excluded from analysis by raising the forward scatter threshold. The percentage of cells in the sub G_0_/G_1_ phase, apoptotic fraction, was quantified using CellQuest software (Becton Dickinson). The distribution of cells in G_0_/G_1_, S, G_2_/M phases was determined using ModFit LT cell cycle analysis software (Becton Dickinson). Results were expressed as a mean ± SD of three experiments performed in triplicate.

### Western Blot Analyses

Treated cells were harvested and disrupted by freeze−thawing in RIPA buffer (50 mM Hepes, 10 mM EDTA, 150 mM NaCl, 1% NP-40, 0.5% sodium deoxycholate, 0.1% SDS, pH 7.4), supplemented with protease inhibitors cocktail (Sigma-Aldrich). Cell debris was removed by centrifugation at 4°C and the supernatant protein concentration was determined, according to the Bio-Rad Protein assay (Biorad Laboratories, CA, USA). Total proteins (30 µg) were separated by SDS-PAGE under denatured reducing conditions. Separated proteins were then transferred to nitrocellulose membranes and blocked for 3 h with a solution containing 3% BSA in 50 mM Tris, 200 mM NaCl, 0.1% Tween 20, before incubation at 4°C overnight with the different primary antibodies (diluted 1∶1000) against specific proteins. After washing, the membranes were incubated with an appropriate peroxidase-conjugate secondary antibodies at room temperature (1 h). Immunoreactive protein bands were detected by enhanced chemiluminescence reagent (ECL, Rockford, USA), according to the manufacturer’s instructions. Quantitative densitometry analyses were performed using a Gel Doc 2000 system (Biorad Laboratories, CA, USA).

### Statistical Analysis

All the reported data represent the mean ± standard deviation (SD) of at least two independent experiments, performed in triplicate. Data were statistically compared by t-test; in that aim we checked for normal distribution of data and comparable variance among the groups compared. The statistical significance of DNA content of hypodiploid nuclei was examined in the two-way analysis of variance (ANOVA) with Bonferroni post-test analysis.

## Results

### Screening for Ligand-HSP90 Complex Formation by Surface Plasmon Resonances

**Figure 2:**
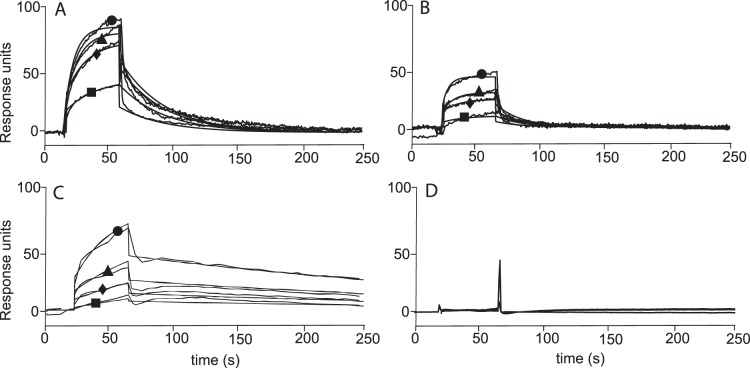
SPR results. Sensorgrams obtained by injecting 25(▪), 50 nM (♦), 250 nM (▴) and 1 µM (•) of geraniin (**A**), compound **1** (**B**), 17-AAG (**C**), and HA (**D**) on immobilized Hsp90α.

Putative interactions of different plant-derived phenolic compounds with Hsp90α were evaluated by a SPR-based approach [27]. Natural compounds belonging to different polyphenol classes (38 flavonoids, 12 coumarins and 4 tannins) were assayed for their binding affinity for the chaperone (Figure 1). The geldanamycin derivative 17-AAG, one of the best characterized Hsp90 inhibitors, was used as a positive control, whereas the clerodane diterpene hardwickiic acid (HA) (Figure S1), was selected as a negative control on the basis of our previous observations [28]. In Figure 2 some of the obtained sensorgrams are reported. Eight out of the 54 tested compounds interacted with immobilized Hsp90α, as inferred by the concentration-dependent responses, and by the clearly discernible exponential curves, during the association and dissociation phases. None of the tested coumarins was found to interact with the protein, whereas several of the tested flavonoids and tannins were able to bind to Hsp90α.

To measure the kinetic and thermodynamic parameters for each complex formation, the sensorgrams were fitted to a single−site bimolecular interaction model (A+B = AB); each constant was calculated fitting at least 12 curves, obtained by injecting the different compounds three times at four different concentrations, ranging from 0.025 µM to 1 µM (Table 1). Among the tested compounds, geraniin showed the highest affinity for the immobilized Hsp90α (Figure 2A). A *K*_D_ of 415±27 nM was measured for the Hsp90α/geraniin complex, comparable to that measured for the Hsp90α/17-AAG complex (388±89 nM).

**Table 1:** Thermodynamic constants measured by SPR for the interaction between tested compounds and immobilized HSP90α.

Compound	K_D_ (nM)
17 AAG	388±89
**54**	415±27
**5**	748±45
**1**	973±97
**34**	1820±242
**6**	2215±351
**2**	4831±515
**4**	5332±862
**19**	8751±735
**HA**	No binding

### Inhibition of Hsp90α by Geraniin

**Figure 3:**
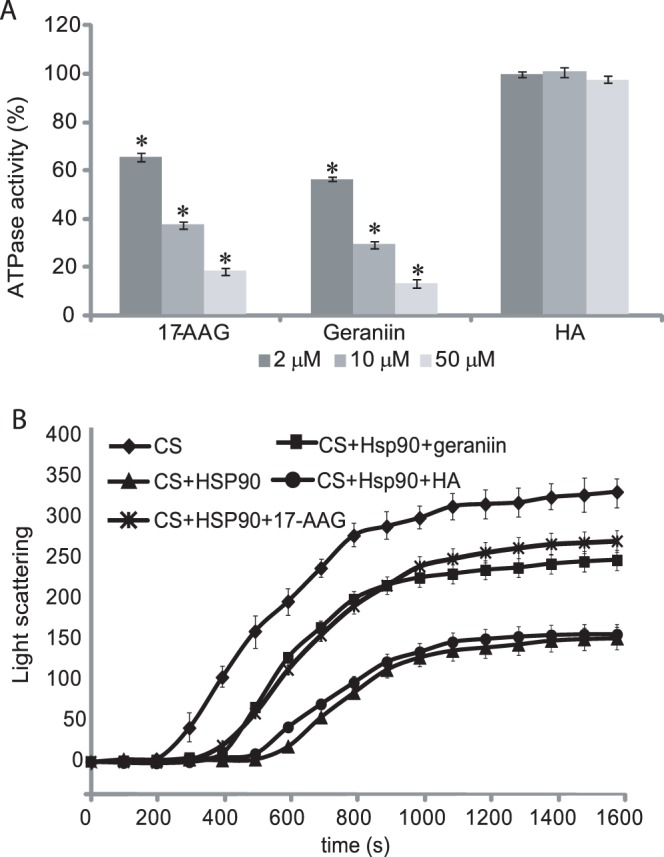
Inhibition of Hsp90α activity. ATPase activity of the chaperone was evaluated in the presence of different concentrations of geraniin, 17-AAG and HA (**A**). Data are reported as the residual ATPase activity (%) compared to that observed for an untreated sample. Data are the mean of three independent experiments performed in triplicate and were analyzed by t test (HA vs testing compounds): The error bar represents the standard deviation of nine measurements, while * indicates significance at P<0.01. Aggregation kinetics of citrate synthase (CS) at 43°C determined by light scattering **(B**)**.** The spontaneous aggregation of CS at 43°C (♦) and the aggregation of CS at 43°C in the presence of 0.075 µM Hsp90 α and 0.3 µM ATP (▴), 0.075 µM Hsp90 α, 0.3 µM ATP and 0.3 µM geraniin (▪), or 0.075 µM Hsp90α, 0.3 µM ATP and 0.3 µM HA (•) are shown. Kinetics traces reported are the averages of three separated measurements; the error bar represents the standard deviation of three measurements.

The elevated affinity towards Hsp90α measured for geraniin, prompted us to investigate further the ability of this ellagitannin to affect different biological activities of the chaperone. Firstly, we evaluated the effects of different concentration of geraniin on the ATPase activity of Hsp90α compared to those produced by 17-AAG or HA, selected as a positive and a negative control, respectively. Geraniin was found to inhibit Hsp90α ATPase activity in a dose−dependent manner, with an inhibitory efficiency similar to that measured for 17-AAG (Figure 3 A).

The next question we addressed was to establish whether geraniin−dependent ATPase inhibition could compromise the chaperone activity of Hsp90α, which was evaluated by monitoring the citrate synthase (CS) thermal induced aggregation in the presence of Hsp90α, with or without geraniin. Upon incubation at elevated temperatures, CS underwent partial unfolding resulting in a quantitative protein aggregation; the presence of stoichiometric amounts of Hsp90α changed the aggregation kinetics significantly. When a four−fold molar excess of geraniin was added to the reaction, the curve slope clearly increased, indicating that geraniin, interacting with Hsp90α, affects its affinity towards denatured CS (Figure 3 B). Anti−chaperone activity of the tannin resembled that observed performing the same experiment using a four−fold molar excess of 17-AAG, whereas the addition of the same amount of HA did not perturb the CS+Hsp90α curve.

### Study of Hsp90α/geraniin Interaction

**Figure 4:**
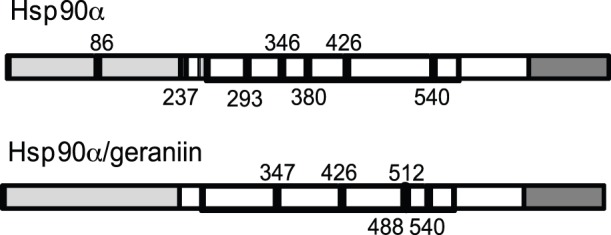
Schematic representation of the results obtained from limited proteolysis experiments. The preferential cleavage sites detected on recombinant Hsp90α and on the Hsp90α/geraniin complex are in black. The Hsp90α *N*-terminal domain is highlighted in light grey, the middle domain is boxed and the *C*-terminal domain is highlighted in grey.

In an effort to identify the Hsp90α region involved in geraniin binding, a limited proteolysis−mass spectrometry−based strategy was used for the structural analysis of the Hsp90α/geraniin complex. This approach is based on the evidence that exposed, weakly structured, and flexible regions of a protein can be recognized by a proteolytic enzyme [29], [30]. The proteolytic patterns obtained in the experiments performed on Hsp90α and on the Hsp90α/geraniin complex, using trypsin or chymotrypsin as proteolytic agents, are summarized in Figure 4. Comparison of the differential proteolytic pattern obtained from the digestion of native Hsp90α or of the Hsp90α/geraniin complex confirmed a direct interaction between geraniin and the chaperone. In addition, we observed that peptide bonds following Arg86, Lys237, Lys293, and Tyr380, preferential cleavage sites of the native chaperone in the absence of geraniin, were protected in the complex. Conversely, following interaction with geraniin, peptide bonds located at the level of Lys488 and Lys512 became susceptible to enzymatic hydrolysis. These data suggest that binding of geraniin to Hsp90α induced significant conformational changes of its three−dimensional structure. Specifically, the observed overall protection of the N−terminal region from proteolysis indicated that this is the protein portion g004mainly involved in interaction with geraniin.

### Effect of Geraniin on Cancer Cell Viability

**Figure 5:**
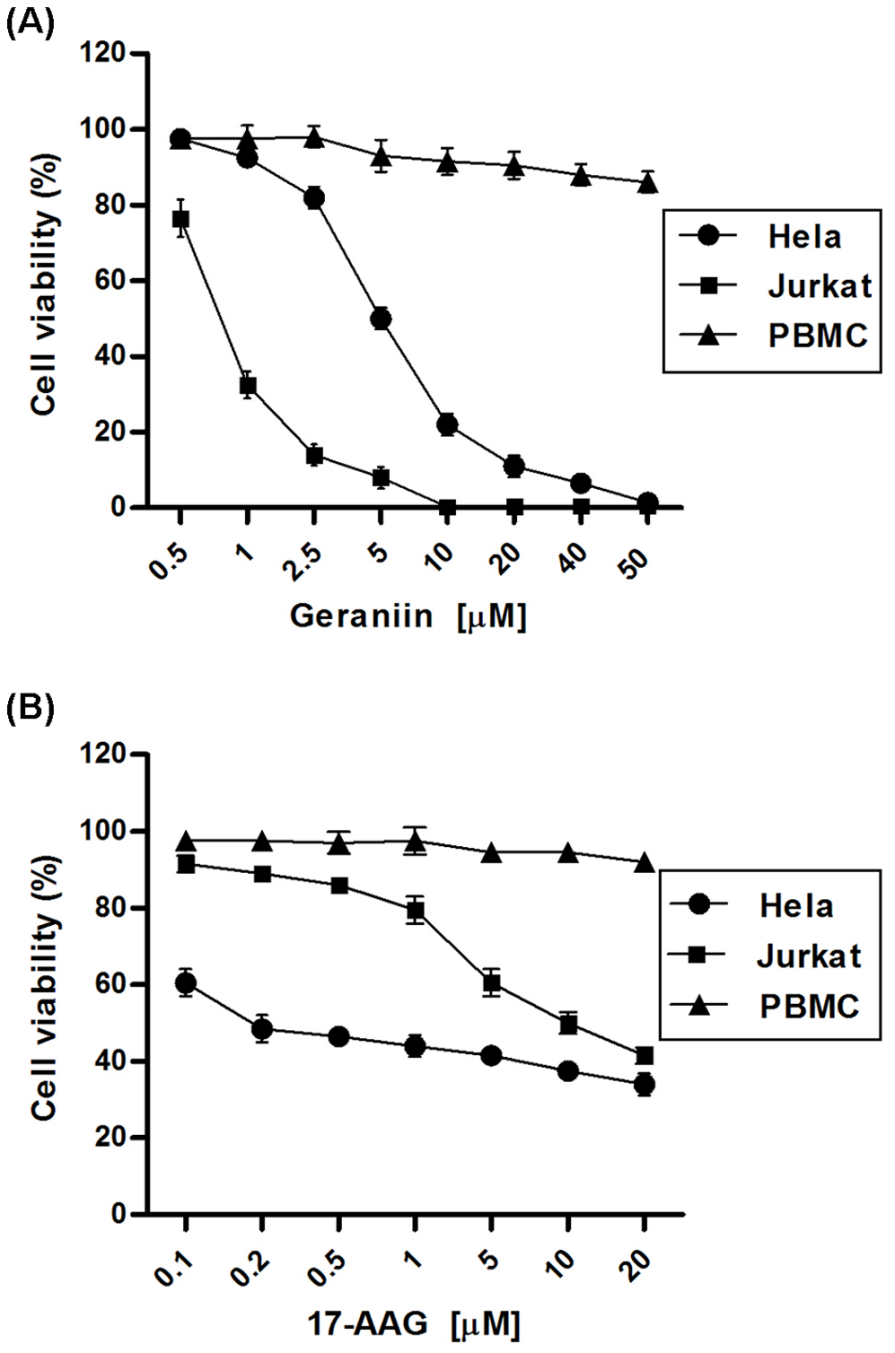
Cell viability (%) of cancer cells and normal cells treated for 24 h with geraniin or 17-AAG. Jurkat, HeLa and PBMC cells were incubated for 24(**A**) or 17-AAG (**B**) used in different concentrations (0.5–50 µM) and (0.1–20 µM), respectively, and processed for cell proliferation determination by the MTT assay.

On the basis of the ability of geraniin to bind and inhibit HSP90α activity, we evaluated its potential anti−proliferative or cytotoxic activity in HeLa (epithelial carcinoma) and Jurkat leukemia cancer cell lines. The cancer cell lines and the PBMC (human Peripheral Blood Mononuclear Cells) line were incubated for 24 h with increasing concentrations of geraniin (0.5 µM – 50 µM) or 17-AAG (0.1 µM – 20 µM) and cell viability was determined by MTT proliferation assay. Proliferation of Hela and Jurkat cells was inhibited by geraniin treatment in a concentration-dependent manner, with IC_50_ values of 5.1±0.2 and 0.76±0.1 µM, respectively (Figure 5 A). Under the same experimental conditions, IC_50_ values after 17-AAG treatment were 9.6±0.15 µM in Jurkat and 0.2±0.3 µM in HeLa cell lines (Figure 5 B), in agreement with those reported by Shelton et al. [31] and Bisht et al. [32]. Minimal cytotoxic effects, evaluated by tryptan blue dying, were observed in control PHA-stimulated proliferating PBMC only at high doses of geraniin or 17-AAG (Figure 5).

### Induction of Cell Cycle Arrest and Cell Death in Cancer Cell Lines by Geraniin

To establish the mechanism of action underlying the inhibition of cancer cell viability caused by geraniin treatment, the cell cycle progression of cancer cells and PHA-stimulated PBMC were analyzed by flow cytometry.

**Figure 6:**
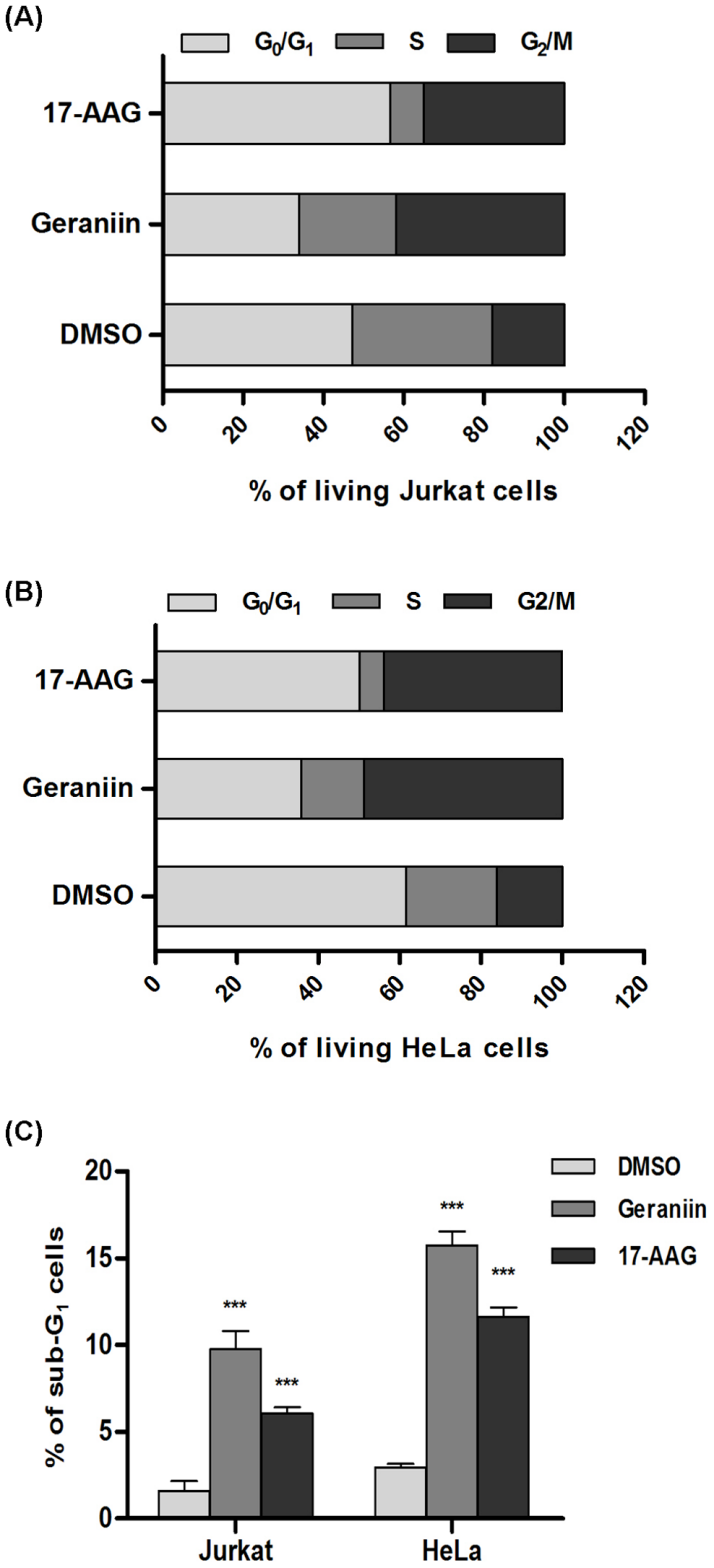
Effects of geraniin and 17-AAG on cell cycle progression. Percentage of cell cycle stages was analyzed by flow cytometry, (**A**) PI-stained viable Jurkat cells treated with DMSO, 0.7 µM geraniin or 10 µM 17-AAG for 24 h. (**B**) PI-stained viable HeLa cells treated with DMSO, 5 µM geraniin or 0.2 µM 17-AAG for 24 h, (**C**) The percentage of hypodiploid cells as treated in **A** and **B**. Results are expressed as means ± SD of three experiments performed in duplicate (***P<0.001).

The Jurkat and HeLa cells were incubated for 24 h with concentrations close to the IC_50_ values of geraniin (0.7 and 5 µM, respectively) and 17-AAG (10 and 0.2 µM, respectively), The effects of geraniin and 17-AAG-treatments on the distribution of non-apoptotic cells differed significantly. In both tumor cell lines, treatment with geraniin caused a G_2_/M arrest (Figure 6 A, B), whereas 17-AAG induced a G_1_ and G_2_/M arrest (Figure 6 A, B), consistent with previous published results [31], [33], [34]. Moreover, following geraniin treatment the percentage of Jurkat and Hela cells with hypodiploid nuclei was 9.75±1.02% and 16.2±0.7%, respectively, whereas following 17-AGG treatment these values were 6.05±0.4% and 11.6±0.5%, respectively. These results indicate a similar pro-apoptotic effect of the two compounds on both the cell lines. Neither geraniin nor 17-AAG caused any pro-death or cytostatic effects in PHA-stimulated proliferating PBMC, since the levels of hypodiploidy/necrosis in geraniin and 17-AAG treated PBMC were 2.6±1.3% and 1.8±0.6%, respectively, very similar to those observed in untreated cells (1.5±0.8%).

### Down−regulation of Hsp90 Client Proteins by Geraniin Treatment

**Figure 7:**
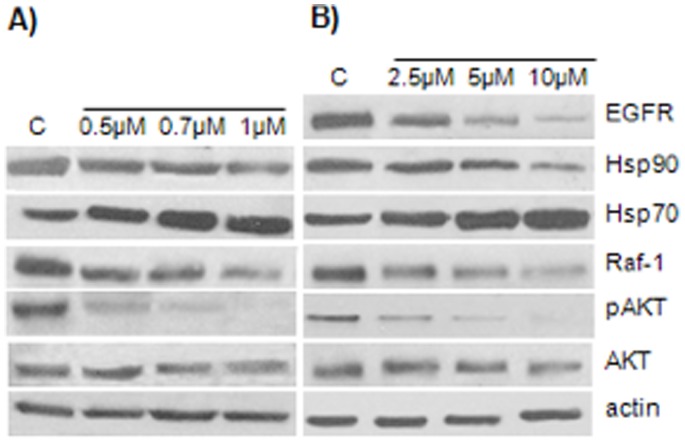
Effect of geraniin on Hsp90 client protein levels. Equal amounts (30 µg) of whole-cell lysates were separated on SDS-PAGE and client proteins were visualized by western blot analysis using specific antibodies. Actin was used as loading control. Total cellular proteins were extracted 24 h after treatment with geraniin (2.5 µM, 5 µM and 10 µM) in Jurkat cells (**A**) or geraniin (1 µM, 0.7 µM and 0.5 µM) in HeLa cells (**B**), ctrl = control cells treated with DMSO). The blots shown are representative of three different experiments with similar results.

Geraniin was tested for its effects on the intracellular levels of Hsp70, Hsp90 and Hsp90 client proteins. HeLa and Jurkat cell lines were treated with a geraniin concentration corresponding to their IC_50_ value and at additional lower and higher concentrations (Figure 7). These treatments caused a dose-dependent decrease in the level of the client proteins c-Raf, pAkt and EGFR, whereas the level of Akt was unaffected. Interestingly, the level of Hsp70 expression was enhanced in both tumor cell lines, in a dose-dependent fashion, with 3.5±0.12-fold increase in Jurkat cells upon 1 µM treatment and 2.7±0.09 fold increase in HeLa cells upon 10 µM geraniin treatment.

## Discussion

Inhibition of HSP90 has received significant attention in cancer research due to its ability to retard or block tumor growth [6]. In this respect, Hsp90 plays a critical role in the maintenance of multiple oncogenic pathways and is required to maintain the folding, stability and functionally active conformation of many aberrant oncoproteins [7]. In healthy cells, Hsp90 is involved in dynamic, low-affinity interactions with a plethora of proteins during folding and maturation; however, in tumor cells, it assists folding of dysregulated oncoproteins and sustains their aberrant activity [4], [5]. Given the diversity of the Hsp90 client proteins involved in critical cellular pathways and processes, inhibition of Hsp90 was predicted to have efficacy in a broad variety of human tumors. However, although several Hsp90 inhibitors have thus far entered into clinical trials, the development of Hsp90 inhibitors has encountered difficulties, including drug solubility and hepatic toxicity [35].

Based on the notion that natural products are compounds pre−optimized by evolution to act against specific biological targets, we performed a structure−based screening of different plant−derived polyphenols to identify new potential Hsp90 inhibitors. By SPR analysis, the tannin geraniin was identified as an efficient ligand of Hsp90α, showing a high affinity for this chaperone, similar to that found for 17-AAG and derivates. The comparison of the HSP90α proteolytic patterns in the presence or absence of geraniin indicated that this compound binds at the N-terminus of the chaperone, as reported for several Hsp90 inhibitors [10]. Through targeting the ATP-binding site of the N-terminal domain, the inhibitors probably prevent Hsp90 from forming a closed N-terminal dimeric state and, consequently, alter the chaperone activity of the molecule [36]. This mechanism was proved for geraniin, which was able to reduce Hsp90 chaperone activity more efficiently than 17-AAG.

The impaired ATPase and chaperone activities of Hsp90, caused by geraniin binding, induce cytotoxic effects in the tumor cell lines tested, with a large percentage of cells containing hypodiploid DNA. These results are in agreement with those reported in human melanoma cells, where geraniin treatment caused apoptosis, through up-regulation of the Fas ligand expression, the activation of caspase-8, the cleavage of Bid, and the induction of cytochrome c release from mitochondria to the cytosol [21]. Our results also revealed that geraniin induces cell cycle arrest in the G_2_/M phase in both cancer cell lines, whereas 17-AAG-treated cells accumulated in G_1_ phase of cell cycle.

Moreover, the geraniin-dependent inhibition of Hsp90α chaperone activity caused a dose-dependent decrease in the level of the oncogenic proteins c-Raf, pAkt and EGFR, further supporting the potential of this compound to interfere with tumor progression. As already reported in other cellular systems, Hsp90 inhibition by geraniin was associated to the up-regulation of Hsp70, as a compensatory mechanism aiding the proper folding and assembling of oncogenic client proteins. Although it is still unclear if the induction of Hsp70 following Hsp90 inhibition attenuates the cytotoxic effects of Hsp90 inhibitors, Hsp70 might also be considered as a pharmacodynamic marker for drug response of HSP90 inhibition [37].

In conclusion, the results presented here, along with the finding that geraniin did not exert any appreciable cytotoxicity in normal cells, encourages further studies on this compound as a promising chemical scaffold for the design of new Hsp90 inhibitors.

## Supporting Information

Figure S1 **Structure of hardwickiic acid.**
(DOCX)

## References

[1] Pratt WB, Toft DO (2003) Regulation of signaling protein function and trafficking by the hsp90/hsp70-based chaperone machinery. Exp Biol Med (Maywood) 228: 111–133. 12563018 10.1177/153537020322800201

[2] Zhang H, Burrows F (2004) Targeting multiple signal transduction pathways through inhibition of Hsp90. J Mol Med 82: 488–499 doi: 10.1007/s00109-004-0549-9 15168026 10.1007/s00109-004-0549-9

[3] Zhang T, Hamza A, Cao X, Wang B, Yu S, et al (2008) A novel Hsp90 inhibitor to disrupt Hsp90/Cdc37 complex against pancreatic cancer cells. Mol Cancer Ther 7: 162–170. 18202019 10.1158/1535-7163.MCT-07-0484

[4] Whitesell L, Lindquist SL (2005) HSP90 and the chaperoning of cancer. Nat Rev Cancer 5: 761–772 doi:10.1038/nrc1716 16175177 10.1038/nrc1716

[5] Holzbeierlein JM, Windsperger A, Vielhauer G (2010) Hsp90: a drug target? Curr Oncol Rep 12: 95–101 doi: 10.1007/s11912-010-0086-3 20425593 10.1007/s11912-010-0086-3

[6] Wang RE (2011) Targeting heat shock proteins 70/90 and proteasome for cancer therapy. Curr Med Chem 18: 4250–4264. 21838681 10.2174/092986711797189574

[7] Solit DB, Rosen N (2006) Hsp90: a novel target for cancer therapy. Curr Top Med Chem 6: 1205–1214 doi: 10.2174/156802606777812068 16842157 10.2174/156802606777812068

[8] Trepel J, Mollapour M, Giaccone G, Neckers L (2010) Targeting the dynamic Hsp90 complex in cancer. Nat Rev Cancer 10: 537–549 doi: 10.1038/nrc2887 20651736 10.1038/nrc2887 PMC6778733

[9] Piper PW, Millson SH (2011) Mechanisms of resistance to Hsp90 inhibitor drugs: a complex mosaic emerges. Pharmaceuticals 4: 1400–1422 doi:10.3390/ph4111400 27721330 10.3390/ph4111400 PMC4060131

[10] Neckers L, Workman P (2012) Hsp90 molecular chaperone inhibitors: are we there yet? Clin Cancer Res 18: 64–76. 22215907 10.1158/1078-0432.CCR-11-1000 PMC3252205

[11] Kummar S, Gutierrez ME, Gardner ER, Chen X, Figg WD, et al (2010) Phase I trial of 17-dimethylaminoethylamino-17-demethoxygeldanamycin (17-DMAG), a heat shock protein inhibitor, administered twice weekly in patients with advanced malignancies. Eur J Cancer 46: 340–347 doi: 10.1016/j.ejca.2009.10.026 19945858 10.1016/j.ejca.2009.10.026 PMC2818572

[12] Heath EI, Hillman DW, Vaishampayan U, Sheng S, Sarkar F, et al (2008) A phase II trial of 17-allylamino-17-demethoxygeldanamycin in patients with hormone-refractory metastatic prostate cancer. Clin Cancer Res 14: 7940–7946 doi: 10.1158/1078-0432.CCR-08-0221 19047126 10.1158/1078-0432.CCR-08-0221 PMC3085545

[13] Yin Z, Henry EC, Gasiewicz TA (2009) (−)-Epigallocatechin-3-gallate is a novel Hsp90 inhibitor. Biochemistry 48: 336–345 doi:10.1021/bi801637q 19113837 10.1021/bi801637q PMC2701625

[14] Brandt GE, Schmidt MD, Prisinzano TE, Blagg BS (2008) Gedunin, a novel hsp90 inhibitor: semisynthesis of derivatives and preliminary structure-activity relationships. J Med Chem 51: 6495–6502 doi: 10.1021/jm8007486 18816111 10.1021/jm8007486 PMC2850591

[15] Dal Piaz F, Vassallo A, Chini MG, Cordero FM, Cardona F, et al (2012) Natural iminosugar (+)-lentiginosine inhibits ATPase and chaperone activity of hsp90. PLoS One 7: e43316 doi: 10.1371/journal.pone.0043316 22916240 10.1371/journal.pone.0043316 PMC3423353

[16] Oh SH, Woo JK, Yazici YD, Myers JN, Kim WY, et al (2007) Structural basis for depletion of heat shock protein 90 client proteins by deguelin. J Natl Cancer Inst 99: 949–961 doi: 10.1093/jnci/djm007 17565155 10.1093/jnci/djm007

[17] Okuda T, YoshidaT, Hatano T (1995) Hydrolyzable tannins and related polyphenols. Fortschr Chem Org Naturst 66: 1–117. 8847006 10.1007/978-3-7091-9363-1_1

[18] Ito H (2011) Metabolites of the ellagitannin geraniin and their antioxidant activities. Planta Med 77: 1110–1115 doi: 10.1055/s-0030-1270749 21294073 10.1055/s-0030-1270749

[19] Clifford MN, Scalbert A (2000) Ellagitannins, occurrence in food, bioavailability and cancer prevention. J Food Sci Agric 80: 1118–1125.

[20] Yang Y, Zhang L, Fan X, Qin C, Liu J (2012) Antiviral effect of geraniin on human enterovirus 71 *in vitro* and *in vivo* . Bioorg Med Chem Lett 22: 2209–2211. 22342145 10.1016/j.bmcl.2012.01.102

[21] Lee JC, Tsai CY, Kao JY, Kao MC, Tsai SC, et al (2008) Geraniin-mediated apoptosis by cleavage of focal adhesion kinase through up-regulation of Fas ligand expression in human melanoma cells. Mol Nutr Food Res 52: 655–663. 18435487 10.1002/mnfr.200700381

[22] Dal Piaz F, Malafronte N, Romano A, Gallotta D, Belisario MA, et al (2012) Structural characterization of tetranortriterpenes from Pseudrocedrela kotschyi and Trichilia emetica and study of their activity towards the chaperone Hsp90. Phytochemistry 75: 78–89 doi: 10.1016/j.phytochem.2011.12.002 22226245 10.1016/j.phytochem.2011.12.002

[23] Dal Piaz F, Tosco A, Eletto D, Piccinelli AL, Moltedo O, et al (2010) The identification of a novel natural activator of p300 histone acetyltranferase provides new insights into the modulation mechanism of this enzyme. ChemBioChem 11: 818–827 doi: 10.1002/cbic.200900721 20373302 10.1002/cbic.200900721

[24] Mosmann T (1983) Rapid colorimetric assay for cellular growth and survival: application to proliferation and cytotoxicity assays. J Immunol Methods 65: 55–63 doi: 10.1016/0022-1759(83)90303-4 6606682 10.1016/0022-1759(83)90303-4

[25] Jakob U, Lilie H, Meyer I, Buchner J (1995) Transient interaction of Hsp90 with early unfolding intermediates of citrate synthase. J Biol Chem 270: 7288–7294. 7706269 10.1074/jbc.270.13.7288

[26] Nicoletti I, Migliorati G, Pagliacci MC, Grignani F, Riccardi C (1991) A rapid and simple method for measuring thymocite apoptosis by propidium iodide staining and flow cytometry. J Immunol Methods 139: 271–279. 1710634 10.1016/0022-1759(91)90198-o

[27] Cooper MA (2003) Label-free screening of bio-molecular interactions. Anal Bioanal Chem 377: 834–842 doi: 10.1007/s00216-003-2111-y 12904946 10.1007/s00216-003-2111-y

[28] Faiella L, Dal Piaz F, Bisio A, Tosco A, De Tommasi N (2012) A chemical proteomics approach reveals Hsp27 as a target for proapoptotic clerodane diterpenes. Mol Biosyst 8: 2637–2644 doi: 10.1039/c2mb25171j 22802135 10.1039/c2mb25171j

[29] Orrù S, Dal Piaz F, Casbarra A, Biasol G, De Francesco R, et al (1999) Conformational changes in the NS3 protease from hepatitis C virus strain Bk monitored by limited proteolysis and mass spectrometry. Protein Sci 8: 1445–1454 doi:10.1110/ps.8.7.1445 10422832 10.1110/ps.8.7.1445 PMC2144388

[30] Giommarelli C, Zuco V, Favini E, Pisano C, Dal Piaz F, et al (2010) The enhancement of antiproliferative and proapoptotic activity of HDAC inhibitors by curcumin is mediated by Hsp90 inhibition. Cell Mol Life Sci 67: 995–1004 doi: 10.1007/s00018-009-0233-x 20039095 10.1007/s00018-009-0233-x PMC11115870

[31] Shelton SN, Shawgo ME, Matthews SB, Lu Y, Donnelly AC, et al (2009) KU135, a novel novobiocin-derived C-terminal inhibitor of the 90-kDa heat shock protein, exerts potent antiproliferative effects in human leukemic cells. Mol Pharmacol 76: 13141322 doi: 10.1124/mol.109.058545 10.1124/mol.109.058545 PMC2784729 19741006

[32] Bisht KS, Bradbury CM, Mattson D, Kaushal A, Sowers A, et al (2003) Geldanamycin and 17-allylamino-17-demethoxygeldanamycin potentiate the in vitro and in vivo radiation response of cervical tumor cells via the heat shock protein 90-mediated intracellular signaling and cytotoxicity. Cancer Res 63: 8984–8995. 14695217

[33] Münster PN, Srethapakdi M, Moasser MM, Rosen N (2001) Inhibition of heat shock protein 90 function by ansamycins causes the morphological and functional differentiation of breast cancer cells. Cancer Res. 61: 2945–2952. 11306472

[34] Arlander SJ, Eapen AK, Vroman BT, McDonald RJ, Toft DO, Karnitz LM (2003) Hsp90 inhibition depletes Chk1 and sensitizes tumor cells to replication stress J Biol Chem. 278: 52572–52577. 10.1074/jbc.M309054200 14570880

[35] Saif MW, Erlichman C, Dragovich T, Mendelson D, Toft D, et al.. (2013) Open-label, dose-escalation, safety, pharmacokinetic, and pharmacodynamic study of intravenously administered CNF1010 (17-(allylamino)-17-demethoxygeldanamycin [17-AAG]) in patients with solid tumors. Cancer Chemother Pharmacol 71: 1345–1355. doi 10.1007/s00280-013-2134-9. 10.1007/s00280-013-2134-9 23564374

[36] Cunningham CN, Krukenberg KA, Agard DA (2008) Intra- and intermonomer interactions are required to synergistically facilitate ATP hydrolysis in Hsp90. J Biol Chem 283: 21170–21178. 18492664 10.1074/jbc.M800046200 PMC2475720

[37] Modi S, Stopeck AT, Gordon MS, Mendelson D, Solit DB, et al (2007) Combination of trastuzumab and tanespimycin (17-AAG, KOS-953) is safe and active in trastuzumab-refractory HER-2 overexpressing breast cancer: a phase I dose-escalation study J Clin Oncol. 25: 5410–5417. 10.1200/JCO.2007.11.7960 18048823

