# Supplementary material for: Identification of the Plant Compound Geraniin as a Novel Hsp90 Inhibitor
Source: PLoS One. 2013 Sep 16;8(9):e74266. doi: 10.1371/journal.pone.0074266 (PMC3774728; doi:10.1371/journal.pone.0074266)
Supplement: Figure S1 — Structure of hardwickiic acid. (DOCX) [file pone.0074266.s001.docx]

**Figure S1**: Structure of hardwickiic acid
